# Management of Primary Central Nervous System Lymphoma Using Intra-Arterial Chemotherapy With Osmotic Blood-Brain Barrier Disruption: Retrospective Analysis of the Sherbrooke Cohort

**DOI:** 10.3389/fonc.2020.543648

**Published:** 2021-01-20

**Authors:** Christian Iorio-Morin, Gérald Gahide, Christophe Morin, Davy Vanderweyen, Marie-André Roy, Isabelle St-Pierre, Karine Massicotte-Tisluck, David Fortin

**Affiliations:** ^1^Division of Neurosurgery, Department of Surgery, Université de Sherbrooke, Centre de recherche du Centre Hospitalier Universitaire de Sherbrooke, Sherbrooke, QC, Canada; ^2^Department of Diagnostic Radiology, Université de Sherbrooke, Centre de recherche du Centre Hospitalier Universitaire de Sherbrooke, Sherbrooke, QC, Canada

**Keywords:** primary central nervous system lymphoma, methotrexate, intra-arterial chemotherapy, blood-brain barrier disruption, survival

## Abstract

**Background:**

Primary central nervous system lymphomas (PCNSL) are rare and aggressive CNS tumors. Current management involves high-dose methotrexate (HD-MTX) typically administered intravenously (IV), despite the existence of the blood-brain barrier (BBB), which significantly decreases its bioavailability. Cerebral intra-arterial chemotherapy (CIAC) coupled with osmotic BBB disruption (OBBBD) can theoretically circumvent this issue.

**Methods:**

We performed a retrospective analysis of patients with newly diagnosed PCNSL treated with HD-MTX-based CIAC+OBBBD at our center between November 1999 and May 2018. OBBBD was achieved using a 25% mannitol intra-arterial infusion. Patients were followed clinically and radiologically every month until death or remission. Demographics, clinical and outcome data were collected from the medical record. All imaging studies were reviewed for evidence of complication and outcome assessment. Kaplan-Meier analyses were used to compute remission, progression-free survival (PFS) as well as overall survival times. Subgroup analyses were performed using the log rank test.

**Results:**

Forty-four patients were included in the cohort. Median follow-up was 38 months. Complete response was achieved in 34 patients (79%) at a median of 7.3 months. Actuarial median survival and PFS were 45 months and 24 months, respectively. Age, ECOG and lesion location did not impact outcome. Complications included thrombocytopenia (39%), neutropenia (20%), anemia (5%), seizures (11%), stroke (2%), and others (20%).

**Conclusion:**

CIAC using HD-MTX-based protocols with OBBBD is a safe and well-tolerated procedure for the management of PCNSL. Our data suggests better PFS and survival outcomes compared to IV protocols with less hematologic toxicity and good tolerability, especially in the elderly.

## Introduction

Primary central nervous system lymphomas (PCNSL) are considered a rare and aggressive form of central nervous system (CNS) tumors, accounting for 1% of lymphomas and representing 4% of primary brain tumors (1, 2). Confined to the brain, eyes and/or cerebrospinal fluid, these extra nodal non-Hodgkin large B cell lymphomas typically show no evidence of systemic diffusion. Untreated, they present a median survival of only 3 months (3).

One major characteristic of these lesions is their sensitivity to both chemotherapy and radiotherapy (4–8). Indeed, using these two modalities in various combinations, the initial overall response rate is in the range of 70%–80% (9–11). Unfortunately, these remissions are frequently short-lasting and a significant number of patients experience disease relapse months or years after treatment (12). Looking at PCNSL median survival over the last decades, the outcome of patients has significantly improved, doubling from 12.5 months in the 1970s to 26 months in the 2010s. This is only true for patients younger than 70 years, as median survival has not changed in older patients, remaining dismal at around 7 months (13).

Over the years, high-dose methotrexate (HD-MTX) has become the backbone of PCNSL treatment and is recommended by the European Association of Neuro-Oncology (EANO) guidelines (9). Many variations of protocols built around HD-MTX have been designed among which the Nordic protocol (14), the memorial Sloan-Kettering Cancer Center (MSKCC) protocol (14) and the Matrix protocol (15), to name only a few. In most recent protocols, investigators tend to postpone or even avoid the use of radiotherapy; indeed, its use has become controversial with findings of associated severe neuro-toxicity, especially in elderly patients (9, 16, 17). Current regimens typically use a standard intravenous (IV) delivery route bearing no consideration to one of the major hurdles in the treatment of CNS diseases: the blood-brain barrier (BBB). Preventing most drugs from entering into the CNS, the BBB poses several challenges to the treatment of CNS pathologies (18) (19–21). It is presumably the reason behind the fact that most protocols for systemic non-Hodgkin lymphomas such as CHOP or CHOD have a low efficacy in primary CNS lymphomas (9, 14, 22, 23). Although the BBB is partially breached at disease presentation, as exemplified by the florid contrast enhancement, it closes as the disease responds to the first cycles of treatment, re-establishing its impermeability (24, 25). Subsequent cycles of treatment are likely less effective, exposing patients to significant systemic doses for a marginal CNS penetration.

Hence, to alleviate this delivery impediment and increase local drug delivery to the lesions, we have been using cerebral intra-arterial chemotherapy (CIAC) coupled with osmotic blood-brain barrier disruption (OBBBD). We hereby report our single-center experience over 18 years with the CIAC + OBBBD technique using two different HD-MTX protocols.

## Methods

### Study Population and Inclusion Criteria

All patients were treated at the Centre hospitalier Universitaire de Sherbrooke (CHUS) between November 1999 and May 2018. The protocol was approved by the institutional review board (Comité d’éthique de la recherche du CIUSSS de l’Estrie—CHUS, FWA #00005894, and IRB00003849, project 00-13), and informed consent was obtained in accordance to institutional regulation in every patient.

All patients with histologically confirmed primary CNS lymphomas were screened for eligibility. Patients were included if they had a KPS > 40 and measurable disease on the initial contrast-enhanced CT/MR scans. Although we treated both newly diagnosed and relapsing PCNSL patients, only newly diagnosed patients are included in the present study. Prior radiotherapy and chemotherapy were thus not allowed.

### Treatment Protocol

After enrollment and initial evaluation, patients were treated every 4 weeks (1 cycle) for 12 cycles, unless progression was demonstrated on imaging. The procedure was accomplished in a standardized way as described by Fortin et al. (26). Briefly, after general anesthesia, a transfemoral approach was used to catheterize either the right or left internal carotid artery or the dominant vertebral artery, depending on tumor’s location. The catheter was placed at the level of the C1-C2 vertebras for the carotid arteries, and at the level of the C5-C6 vertebra for the dominant vertebral artery. A diagnostic angiogram was systematically obtained prior to treatment.

OBBBD was performed by infusing a 25% mannitol solution in the selected vascular distribution for 30 s at a rate that fills the vascular distribution so as to maximize the contact of the mannitol with the endothelial cells. The rate of infusion, ranging from 3 to 6 mL/s, was carefully selected for each treated vessel to prevent reflux into the common and external carotid arteries. Once determined, the mannitol was infused over a 30 s period. The chemotherapy was then infused *via* the intraarterial (IA) route at a rate calculated to prevent streaming (27).

Because the procedure induces a transitory increase in intracranial pressure, only one vascular distribution was disrupted per monthly session. Thus, in patients with lesions encompassing more than one vascular territory, different vascular distributions were treated alternately from cycle to cycle to cover the entire territory of disease involvement. The treatment proceeded using this approach until a complete response was observed. A complete response (CR) was defined as a complete disappearance of abnormal parenchymal contrast enhancement in patients exempt from dexamethasone, as per the International Primary CNS Lymphoma Collaborative Group Response criteria (12). Once a CR was reached, we started to cycle vessel distributions so that all cerebral vascular territories were covered with an intraarterial infusion for at least one cycle. A total of 12 cycles was targeted.

The presence of a significant mass effect, as exemplified by a closed quadrigeminal cisterna, dilatation of the contralateral ventricular system or uncal herniation, represented an absolute contraindication to OBBBD. Thus, patients presenting with a mass effect deemed excessive (n=20) but otherwise meeting all the inclusion criteria were initially offered CIAC without OBBBD, until the mass effect had subsided.

Two different chemotherapy regimens were sequentially used in this study. High-dose MTX (5 g IA) was the cornerstone of both regimens: the first regimen (1999 to 2007) also included etoposide phosphate (400 mg/m² IV) and cyclophosphamide (660 mg/m² IV), whereas the second regimen (2008 to 2018) also included carboplatin (400 mg/m² IA). Folinic acid rescue was used at 36 h after MTX administration, and filgrastim was routinely administered to patients (5 mcg/kg s/c die) for 7 days.

### Follow-Up

All patients were monitored with complete blood and platelet counts every week. A biochemical, kidney and liver function profile, and electrolyte assessment was requested every 4 weeks. Neurological and general physical examination as well as control MRI were performed before each cycle. Once the 12^th^ cycle was completed and treatment discontinued, patients were followed with a neurological examination and MR scan every 3 months for the first year and every 6 months thereafter.

### Data Collection and Response Assessment

Demographics, clinical data and adverse events were prospectively collected from study entry and at every visit during the study. Adverse events were reclassified and graded at the time of analysis using the Common Terminology Criteria for Adverse Events (CTCAE) Version 5.0. All adverse events of grade 3 or higher as well as all ischemic events (any grade) are reported.

The radiological response and complications reported in this study were assessed post-hoc by an independent neuroradiologist (GG). Response was defined as per the criteria described by Abrey et al. (12). These are a modification of the MacDonald criteria designed specifically for PCNSL. Briefly, a complete response (CR) required the complete disappearance of contrast enhancement on MRI, no evidence of ocular lymphoma, negative CSF cytology, and discontinuation of corticosteroid use for at least 2 weeks prior to the evaluation of response. The time to remission was defined as the first MRI demonstrating CR. Hence, only patients who reached CR were considered in remission.

### Statistical Analysis

Descriptive statistics are presented as median with interquartile range, or as the total number of event (n) with valid percentage. Total n = 44 unless otherwise specified. The primary endpoint of this study was overall survival, with remission induction, PFS, vascular complications and vascular complications used as secondary outcomes. Remission induction, PFS and median survival times were computed using the Kaplan-Meier estimator. The univariate impact of age (< vs ≥ than 70), lesion multiplicity (single vs multiple), lesion location (superficial vs deep), chemotherapy regimen (without vs with carboplatin), mass effect preventing initial OBBBD (yes vs no) and ECOG at presentation (0–1 vs 2 vs 3–4) on these outcomes was assessed using the log rank test after confirming the similarity of the censoring patterns of each distribution. A conditional, forward, stepwise Cox regression analysis was also performed using the aforementioned variables and outcomes. Statistical significance was defined as p <.05. All statistical analyses were performed using IBM SPSS Statistics, version 25 (IBM, Armonk, New York) and figures generated using SPSS Statistics and Adobe Illustrator CS6 (Adobe Systems Inc, San Jose, California).

## Results

**Figure 1:**
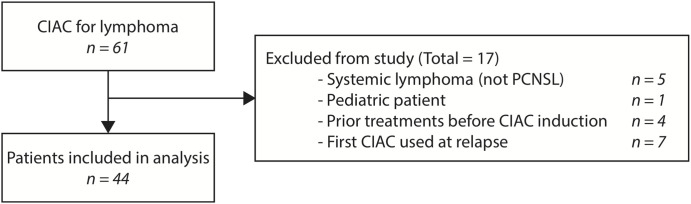
Flow diagram of patients included in the study.

Overall, a total of 61 patients were accrued and received at least one cycle of treatment. Of these, 17 were excluded from the analysis: one pediatric patient, four patients secondarily referred to our institution after having received prior systemic chemotherapy at another, five patients with systemic lymphomas and seven patients initially treated with an IV HD-MTX regimens (**Figure 1**).

The present analysis therefore includes 44 naive patients recruited over 18 years in a single center. Median follow-up was 38 months. From November 1999 to 2007, 15 patients (33%) were exposed to the first regimen whereas 29 patients (67%) were exposed to the HD-MTX and carboplatin regimen after 2007.

### Patients and Tumors Characteristics

The median age of the cohort (25 males and 19 females) was 62 years (IQR: 14). Forty-three patients presented a diagnosis of B-cell lymphomas, whereas one was diagnosed with a T-cell variant. Forty-three patients underwent a biopsy as the diagnostic procedure, and one underwent an open surgical partial resection at another institution. The functional status of the patients at presentation is summarized in **Table 1**.

**Table 1:** Demographics and clinical characteristics.

Median age at diagnosis, years	62.3 (14.0)
Sex Female Male	19 (43%) 25 (57%)
ECOG at enrollment (n = 43) 0 1 2 3 4	2 (5%) 20 (47%) 13 (2%) 7 (16%) 1 (2%)
Median number of lesions at presentation	1.5 (2.0)
Vascular territory supplying lesions Single Multiple	30 (68%) 14 (32%)
Lesion location Superficial Deep Mixed	22 (51%) 16 (36%) 6 (14%)
Bilateral location	7 (16%)
Previous surgical procedure Biopsy Cytoreduction	43 (98%) 1 (2%)
Histologic diagnosis Diffuse large B-cell lymphoma T-cell lymphoma	43 (98%) 1 (2%)

On the initial presenting MR scan, 21 patients displayed a unique lesion (49%), nine patients depicted two lesions (21%), nine patients presented three lesions (19%), two patients presented four lesions (5%), and three patients had more than five lesions (7%), with a maximum of 12 lesions for one patient. In terms of tumor localization with respect to the depth topography of disease involvement, 22 patients (51%) presented with superficial lesions only, whereas 16 (35%) presented with deep lesions and 6 (14%) had mixed localizations. Seven patients displayed a bi-hemispheric lesion crossing the midline *via* the corpus callosum (16%) and 14 patients had multifocal lesions involving at least two vascular territories (32%).

### Response to Treatment and Outcome

**Figure 2:**
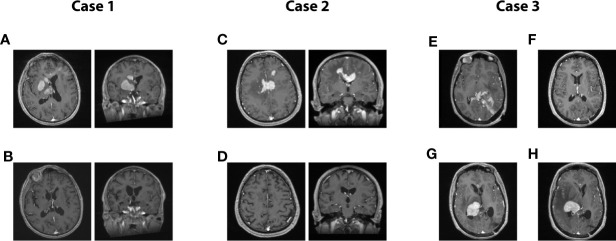
Representative cases from the series. **(A, B)**: Case 1. A 71-year-old man presented with rapidly progressing left-sided hemiparesis combined with a sudden decline in cognitive functioning. On T1-gadolinium enhanced MRI, multiple nodular enhancements are obvious, devoid of significant oedema. Local mass effect can be observed on the ventricular system in axial and coronal acquisitions **(A)**. Same MRI acquisition in axial and coronal after eight cycles of treatment **(B)**. This patient remains disease-free to this date, 18 months after the conclusion of his last cycle. Clinically, he presented a full recovery. **(C, D)**: Case 2. A 62-year-old man presented a gradual deterioration in cognitive functioning over a 2 months interval. Significant slowness in motor initiation and working memory were prominent features on presentation. A T1-enhanced MRI scan revealed multiple nodular enhancing lesions, mostly centered on the corpus callosum, and accompanied by oedema, in axial and coronal **(C)**. T1-enhanced MRI scan in axial and coronal acquired at the conclusion of the treatment plan of 12 cycles, displaying a complete radiological response which was paralleled by a full clinical recovery **(D)**. The patient remains disease-free 7 years after the conclusion of his treatment plan. **(E–H)**: Case 3. A 42-year-old woman presented with mixed aphasia, confusion and severe headaches. What was considered confusion in retrospect was entirely attributable to a receptive speech deficit. She underwent an MRI scan which revealed a diffuse T1-enhancing mass localized in the left parieto-occipital area and diffusely infiltrating the splenium. The mass was partially resected in another center, before she started treatment with our team. The pre-treatment axial T1 MRI is shown in Panel **(E)**, while the post-treatment MRI at the conclusion of eight cycles is shown in Panel **(F)**. The patient was in complete response and had presented a full clinical recovery. She hence decided to stop treatment against our advice. Five months later, she presented with a left-sided deficit prompting an investigation. The T1-enhanced MRI revealed a relapse in disease in the contralateral hemisphere, as displayed on the axial T1-enhanced contrast MRI **(G)**. She resumed treatment and responded for three cycles, only to present a massive progression before the initiation of the 4^th^ cycle **(H)**. The patient then elected to discontinue all treatment, and she passed away 1 month after the relapse.

Overall, a CR was induced in 35 patients (80%). The median time to CR was 7.3 months (IQR: 7.8). Of these 35 patients, 11 are still alive and nine remain disease-free after treatment discontinuation. The two patients that presented a relapse were retreated with the same protocol, and a CR was re-induced. Of the patients who did not obtain a remission (n=9), one is still under treatment (cycle #8), whereas eight deceased from their disease. Representative cases are presented in **Figure 2**.

**Figure 3:**
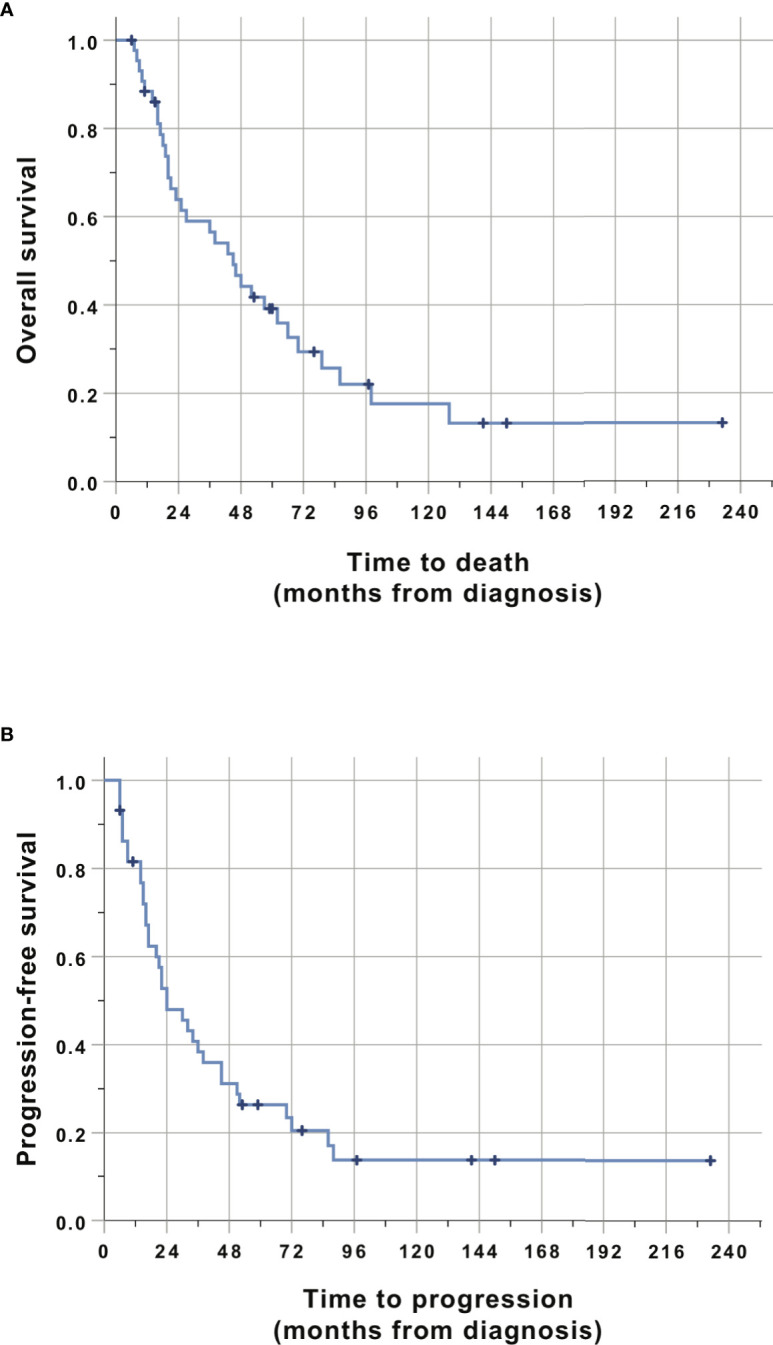
Kaplan-Meier analysis of overall survival **(A)** and progression-free survival **(B)**. Time is relative to the initial diagnosis of PCNSL. Cases were censored at the last clinical follow-up (marked in dark color).

The actuarial median survival (MS) was 45 months (95% CI: 30.1–60.0) for the cohort. Survival was 88%, 64%, 57%, 39%, and 18% at 1, 2, 3,5 and 10 years (**Figure 3** and **Table 2**). The progression-free survival was 24 months (95% CI: 12.5–35.5). The actuarial PFS was 82%, 53%, 38%, 26%, and 14% at 1, 2, 3, 5, and 10 years. Three patients died of unrelated causes after having obtained a CR and were still without signs of progression at the moment of their death. They died at 7, 8, and 23 months, respectively, after the completion of their treatment. All the other patients who died did so from tumor relapse or progression.

**Table 2:** Tumor control and survival following CIAC with OBBBD.

Median follow-up from diagnosis, months	38 (46.4)
Median number of cycles	10 (4)
Chemo regimen Carbo + MTX Other	29 (66%) 15 (34%)
Crude remission	34 (79%)
Median time to remission, months	7.3 (7.8)
Actuarial PFS 1-year PFS 2-year PFS 3-year PFS 5-year PFS 10-year PFS Median PFS	82% 53% 38% 26% 14% 24 months (95% CI: 12.5–35.5)
Crude death	32 (72%)
Actuarial survival 1-year survival 2-year survival 3-year survival 5-year survival 10-year survival Median survival	88% 64% 57% 39% 18% 45 months (95% CI: 30.1–60.0)

**Figure 4:**
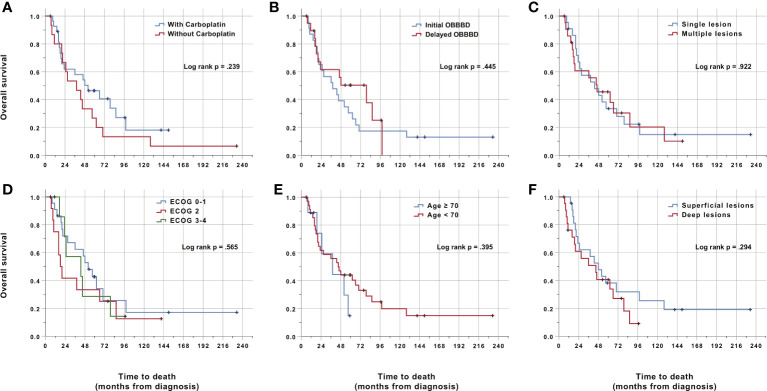
Kaplan-Meier analysis of overall survival. Time is relative to the initial diagnosis of PCNSL. Cases were censored at the last clinical follow-up (marked in dark color). There was no statistically significant difference in survival between any subgroup in the univariate analysis. **(A)** Stratification by chemotherapy regimen. Fifteen patients were treated between 1999 and 2007 with HD-MTX, etoposide phosphate and cyclophosphamide (red line, one censored case). Twenty-nine patients were treated between 2008 and 2018 with carboplatin in addition to the previous drugs (blue line, 10 censored cases). One patient was treated with both regimens and was excluded from the analysis. **(B)** Stratification by initial use of OBBBD. Twenty-three patients had OBBBD performed during their first cycle (blue line, three censored cases). Twenty patients received their first cycle without OBBBD because of significant mass effect precluding safe OBBBD (red line, eight censored cases). All patients eventually received OBBBD. The first cycle protocol was missing for one patient, which was excluded from this analysis. **(C)** Stratification by single (blue line, 22 patients with five cases censored) vs multiple lesions (red line, 22 patients with seven cases censored). **(D)** Stratification by ECOG at the time of first treatment. Two patients with an ECOG of 0 were pooled with 20 patients with an ECOG of 1 (blue line, four censored cases). Thirteen patients had an ECOG of 2 (red line, three censored cases). Seven patients with an ECOG of 3 were pooled with one patient with an ECOG of 4 (green line, two censored cases). ECOG data was missing for one patient. **(E)** Stratification by age ≥ 70 (blue line, nine patients, three censored cases) vs < 70 (red line, 35 patients, nine censored cases). **(F)** Stratification by deep (involving the corpus callosum, thalamus or basal ganglia, red line) vs superficial (blue line) lesion location. Each group had 22 patients with six censored cases.

Subgroup analyses were performed to identify predictors of outcome. No variable was independently associated with either a poor survival or PFS (**Figure 4**). There was a trend towards better survival and PFS when carboplatin was added to the regimen (**Figure 4A**), although this did not reach statistical significance (p = .239) either in the univariate or multivariate analysis. The ECOG at the time of treatment, the age of the patient, the number of lesions and lesion location did not impact survival or PFS. Furthermore, the presence of mass effect requiring the initiation of the treatment using CIAC only, without OBBBD did not affect outcomes either (**Figure 4B**). Multivariate analyses using Cox regression did not highlight any variable predictive of either survival or PFS (data not shown).

### Complications and Adverse Reactions

Complications are divided and presented as such: vascular complications related to the process of intraarterial infusion and OBBBD, peri-procedural seizures, hematological complications and other adverse events (**Table 3**). Only grades 3 and 4 toxicities are reported for hematologic complications. There was no instance of grade 5 toxicity.

**Table 3:** Complications following CIAC with OBBBD.

**Ischemic complications** Grade 1 (asymptomatic lacunar stroke) Grade 2 (transient ischemic attack) Grade 3 (stroke) Grade 4 (life-threatening stroke) Grade 5 (death)	**5 (11%)** 3 (7%) 1 (2%) 1 (2%) 0 0
**Seizures** Grade 1 (focal seizure) Grade 2 (generalized seizure) Grade 3 (multiple, medication-resistant seizures) Grade 4 (life-threatening seizure) Grade 5 (death)	**5 (11%)** 0 4 (9%) 0 1 (2%) 0
**Hematological complications** **Anemia** Grade 3 (Hb < 80 g/L, or transfusion) Grade 4 (life-threatening consequences) Grade 5 (death) **Thrombocytopenia** Grade 3 (Plt 25–50 x 10^9^) Grade 4 (Plt < 25 x 10^9^) **Neutropenia** Grade 3 (0.5-1 x 10^9^) Grade 4 (< 0.5 x 10^9^)	**2 (5%)** 2 (5%) 0 0 **17 (39%)** 10 (23%) 7 (16%) **9 (20%)** 8 (18%) 1 (2%)
**Other complications** ** Anaphylaxis to carboplatin** ** **Grade 3 (symptomatic bronchospasm) **Sepsis** ** **Grade 4 (life-threatening) ** Lung infection (pneumonia)** Grade 3 (IV antibiotics indicated) ** Thromboembolic event (pulmonary embolism)** Grade 3 (urgent medical intervention indicated)	2 (5%) 1 (2%) 1 (2%) 1 (2%)

Bolded values indicate overall rate.

No complications, such as arterial dissection or puncture, were identified on angiography. Five (11%) vascular ischemic events related to the treatment were observed on post-procedure MRI (**Table 3** and **Supplementary Table 1**). Only one of these produced a grade 3, permanent deficit (transcortical aphasia from a left internal carotid stroke). All other events were either grade 2 (one transient ischemic attack) or grade 1 (three asymptomatic lacunar strokes).

Five patients (11%) presented at least one episode of per-procedural seizure during the perfusion of MTX (four grade 2 and one grade 4). In these instances, the perfusion was discontinued, and a bolus of propofol was administered to the patient. Once the seizure was controlled, MTX perfusion was resumed. One patient (2%) developed a status epilepticus that was eventually controlled but required a 3 days intubated stay in the ICU (grade 4 complication).

Hematologic complications are summarized in **Table 3**. Thrombocytopenia was, by far, the most frequent hematologic adverse event, with 10 and seven patients suffering from grades 3 (23%) and 4 (16%), respectively. We also observed 8 instances of grade 3 neutropenia (18%), and one grade 4 event (2%). Anemia was uncommon with only two patients (5%) presenting a grade 3 event (Hb < 80 g/L).

Two cases of anaphylactic reaction to carboplatin infusion were observed (grade 3). Although these reactions were not severe, they prompted desensitization procedures for subsequent cycles. One case of severe sepsis (grade 4), one pneumonia (grade 3) and one instance of pulmonary embolism (grade 3) were finally observed (**Table 3**).

## Discussion

Care and prognosis for PCNSL has significantly changed in the last decades. Indeed, median survival has nearly increased by a 2-fold factor, passing from 12.5 months in the 1970s to 26 months, in the 2010s (3). This improvement has mostly been realized by introducing HD-MTX as the mainstay of the induction phase. Different combinations have been proposed to accomplish the induction, all articulated around HD-MTX, followed by a maintenance phase. This maintenance phase is highly variable, but most commonly has consisted of whole-brain radiation therapy (28). Interestingly, although this treatment modality has been shown to increase PFS, its impact on survival is still disputed (28). More distressing, however, are the severe signs of neurotoxicity that have been observed with its use (29). This neurotoxicity can be delayed, and is observed mostly in long term survivor patients, older than 60 year old (30, 31). Some studies even estimated the incidence of severe cognitive deficits to be close to 100% in these patients (17). Recently, some investigators even questioned the efficacy of radiotherapy in prolonging survival in PCNSL patients (4). Hence, an approach based on HD-MTX poly-chemotherapy for the induction as well as for the maintenance phase is contemplated and should be privileged.

This leaves us with one major predicament: the presence of the BBB. As hinted by the typically florid contrast MRI enhancement at disease presentation, the BBB is not so much an obstacle at treatment initiation as it is at maintenance, when contrast enhancement disappears (18). As soon as the BBB regains its impermeability, subsequent chemotherapy cycles gain less entry, and are likely less effective. Moreover, this disease is infiltrative and often multifocal at presentation, thereby suggesting that tumor cells can be found at a distance from enhancing nodules and consequently of barrier breach. This has led some authors to recommend using T2/FLAIR anomalies in response assessment as a surrogate for tumor in addition to the enhancing lesions where BBB is disrupted (12). This unique situation might contribute to the incomplete eradication of the disease in some patients and play a role in PCNSL relapse. To counter the delivery limitation of therapeutics to the CNS, we advocate the deployment of a delivery strategy in an attempt to bypass the BBB as an adjunct to chemotherapy infusion (18, 19). Henceforth the use of CIAC infusion combined with OBBBD.

Neuwelt et al. were pioneers in the field of BBB modification using the osmotic approach. They have published an extensive body of work in this field (32–34). This group reported a median overall survival of 3.1 years using IA HD-MTX, combined to etoposide, cyclophosphamide and/or procarbazine (21). They have also deployed every effort to study the neuro-cognitive impact of repeated OBBBD procedures combined to HD-MTX poly-chemotherapy treatment in PCNSL patients. Indeed, following 26 long-term survivors out of 112 patients treated with intra-arterial MTX and OBBBD since 1982 (median follow-up of 12 years), they found improvement or stabilization of the cognitive functioning in every patient, compared to the pre-treatment evaluation (17). This was observed through detailed and thorough neuro-cognitive assessments. From this data, we can hypothesize that repeated OBBBD treatments combined to IA HD-MTX are unlikely to produce significant neurotoxicity.

The overall safety of this treatment strategy has already been established and reported on. Doolittle et al. (20). reported on the overall complication rate in the OBBBD treatments of 221 patients from five centers over 3 years. A vascular complication rate of 5% (asymptomatic subintimal tear) was then reported. This report, however, did not systematically review, as we did, the entirety of the MR scans obtained in all the treated patients. These investigators also mention four cases of brain herniation related to the treatment. OBBBD has been shown to increase brain extracellular water content by approximately 1.5%, thus potentially tipping the balance for patients already in severe intracranial hypertension (32). This is unfortunately most likely what happened in these four patients. We did not observe a single instance of herniation in our series. The stringent exclusion criteria for significant mass effect that we use appears to be adequate in preventing these cases. Hence, the high number of patients (n=20) that were started on CIAC without adjunctive OBBBD for the initial cycles. These were later converted to OBBBD once a decrease in mass effect was observed and deemed safe (usually after a single CIAC treatment cycle). Interestingly, when analyzed as a subgroup, these patients initially started with CIAC without OBBBD were not adversely affected in terms of OS or even PFS.

In terms of vascular complications related to the OBBBD and CIAC, not only did we review all the angiographies, but also did we review all MR scans. We did not observe a single case of sub-intimal tear in this series. Looking at the incidence of MR changes associated with new clinical symptoms post treatment, we found five cases of vascular complications related to the treatment (11%) of which only one was permanent (2%). This is obviously far from negligible, but reasonable in the context of an aggressive treatment for a lethal disease. Indeed, when looking at the complications in the present series, our results compare favorably to those obtained by Ferreri et al., reporting on the use of the MATrix regimen (15). This group observed a 6% incidence (13 patients/221) of grade 5 toxicity (death related to treatment toxicity) with this regimen, whereas we had no such occurrence.

Our results compare favorably with current series recently published, while permitting us to avoid radiotherapy. With a MS of 45.0 months, our results are on par with the results obtained by the EORTC, using high-dose MTX+ WBRT (35). Recently, Da Broi et al. reported the results of 57 patients treated over 12 years with chemotherapy (14). Overall, a median OS of 35.4 months, and a PFS of 15.7 months were observed. Looking at a comparison between the use of the Nordic protocol vs the Memorial Sloan-Kettering Cancer Center (MSKCC) protocol ± rituximab, the investigators found the best regimen to be MSKCC + rituximab, with a median OS of 46 months. The MSKCC without rituximab produced a median OS of 15.2 months, whereas the Nordic protocol presented a median OS of 30.9 months.

This is in keeping with recent reports suggesting that rituximab, an anti-CD20 monoclonal antibody, clearly appears beneficial in the outcome of PCNSL patients (35, 36). Muldoon et al. have shown in a mouse model of PCNSL that rituximab was effective in the initial phase of treatment of the disease, when administered intravenously with a capacity to marginally cross the BBB (37). However, as it is a high-molecular weight compound, this monoclonal antibody did gain being administered after a OBBBD, in terms of delivery, especially in the brain distant to the tumor, where the barrier breach is modest or inexistent (4-fold delivery compared to IV). Hence, we feel rituximab should be incorporated to future PCNSL regimen, and it would probably gain from an adjunctive OBBBD procedure.

Finally, we need to emphasize the fact that age was not a predictive factor for survival and response in this series, thus hinting at the fact that this regimen is well tolerated, even by aged patients. CIAC + OBBBD procedures are not riskier in this subset of patients. To our knowledge, this is the first study reporting similar outcome results for older patients. Likewise, an initially dismal clinical condition as reflected by a poor ECOG score was in no way predictive of an adverse outcome and should not influence on the aggressiveness of the treatment. PCNSL patients have the potential to present a significant cognitive recovery pending a good response. The same can be said of the initial MR images, keeping in mind that deep structures, bilateral and multifocal involvement are by no mean associated with an adverse outcome, at least in our series.

### Limitations

The biggest limitation of our series is its retrospective nature and inherent selection bias that might have occurred. Contrary to many prospective studies, however, age and ECOG were not considered exclusion criteria. The external validity of our findings is limited by the single-center design of the study. Because of the rarity of PCNSL, patients were also enrolled over the course of 18 years, during which treatments outside of our protocol have changed significantly. Comparison of the 18-year cohort to prospective studies performed during a specific era must therefore be made carefully.

## Conclusion

CIAC HD-MTX-based protocols with OBBBD is a safe and well-tolerated procedure for the management of primary CNS lymphoma. Our single center data suggests better PFS and survival outcomes compared to IV protocols, with less hematologic toxicity and good tolerability, especially in the elderly. In terms of response, elderly patients were not adversely affected, suggesting that this regimen is effective in this sub-groups of patients. The next iteration of our protocol will continue to use CIAC delivery, but also include rituximab to further improve on those results. This strategy should be formally assessed in a randomized controlled trial with special emphasis on elderly patients, for whom options are lacking.

## Data Availability Statement

The datasets generated for this study are available on request to the corresponding author.

## Ethics Statement

The studies involving human participants were reviewed and approved by Comité d’éthique de la recherche du CIUSSS de l’Estrie—CHUS, FWA #00005894 and IRB00003849. The patients/participants provided their written informed consent to participate in this study.

## Author Contributions

Study design: DF, CI-M. Data collection: CI-M, GG, CM, DV, M-AR, IS-P, KM-T, DF. Manuscript redaction: DF, CI-M. Manuscript revision: GG, CM, DV, MA-R, IS-P, KM-T. All authors contributed to the article and approved the submitted version.

## Funding

This study was funded from general funds provided to the neuro-oncological laboratory by the Fondation du Centre Hospitalier Universitaire de Sherbrooke and the Fondation de l’Université de Sherbrooke.

## Conflict of Interest

The authors declare that the research was conducted in the absence of any commercial or financial relationships that could be construed as a potential conflict of interest.
