# Supplementary material for: Management of Primary Central Nervous System Lymphoma Using Intra-Arterial Chemotherapy With Osmotic Blood-Brain Barrier Disruption: Retrospective Analysis of the Sherbrooke Cohort
Source: Front Oncol. 2021 Jan 20;10:543648. doi: 10.3389/fonc.2020.543648 (PMC7855856; doi:10.3389/fonc.2020.543648)
Supplement: Supplementary file 1 [file Table_1.docx]

**SUPPLEMENTARY TABLE 1**

| **Symptomatic Ischemic complications following intraarterial chemotherapy**  **In 11.4% of patients (n=5)** | | | | |
| --- | --- | --- | --- | --- |
| Type | Number | Location | Symptoms | Recovery |
| Lacunar strokes | N= 3 (6.8%) | Vertebrobasilar  N=2 (4.5%) | Gait instability, diplopia and vertigo | Subsided over 2 months |
|  |  |  | Dysmetria | Resolution in 2 weeks |
|  |  | Head of the right caudate nucleus N=1 (2.3%) | Left brachio-facial seizures | Resolution in 2 months |
| Transient Ischemic Attack | N=1 (2.3%) | Right Internal Carotid Artery | Left hemiplegia | Resolution in a few hours |
| Stroke | N=1 (2.3%) | Left Internal Carotid Artery | Transcortical Aphasia | Permanent |
